# Corrigendum to: “Removing ECG Artifact from the Surface EMG Signal Using Adaptive Subtraction Technique” published in J Biomed Phys Eng 2014; 4(1):31-38

**Published:** 2015-06-01

**Authors:** S. Abbaspour, A. Fallah

**Affiliations:** 1 Department of Biomedical Engineering, Amirkabir University of technology, Tehran, Iran

In the version of this article initially published, there was an error in figure 6. The corrected figure 6 was as below.

**Figure 6:**
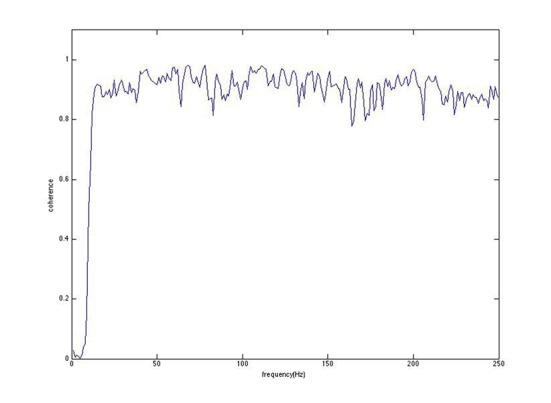
Coherence of clean EMG and cleaned EMG signals.

This error has been corrected in the PDF version of the article. 

